# Nanotechnology-based approaches in the fight against SARS-CoV-2

**DOI:** 10.3934/microbiol.2021023

**Published:** 2021-10-12

**Authors:** Alrayan Abass Albaz, Misbahuddin M Rafeeq, Ziaullah M Sain, Wael Abdullah Almutairi, Ali Saeed Alamri, Ahmed Hamdan Aloufi, Waleed Hassan Almalki, Mohammed Tarique

**Affiliations:** 1 Molecular Medicine Genetics, Department of Oncology and Human Metabolism, the Medical School, University of Sheffield, Beech Hill Road, Sheffield, S10 2RX, United Kingdom; 2 Department of Pharmacology, Faculty of Medicine, Rabigh. King Abdulaziz University. Jeddah, 21589, KSA; 3 Department of Microbiology, Faculty of Medicine, Rabigh. King Abdulaziz University, Jeddah, KSA 21589; 4 Department of Respiratory Services, Ministry of National Guard Hospital and Health Affairs (MNGHA) P.O. box 22490, kingdom of Saudi Arabia; 5 Molecular Pathology Lab Department of Pathology and Laboratory Medicine, Ministry of National Guard Hospital and Health Affairs (MNGHA), P.O. box 22490, Kingdom of Saudi Arabia; 6 Department of Pathology and Laboratory Medicine, Ministry of National Guard-Health Affairs P.O. box 22490, Kingdom of Saudi Arabia; 7 Department of Pharmacology and Toxicology, Umm Al-Qura University, Makkah, Kingdom of Saudi Arabia; 8 Center for Interdisciplinary Research in Basic Sciences, Jamia Millia Islamia, Jamia Nagar, New Delhi-110025, India

**Keywords:** COVID-19, SARS-CoV-2, nanotechnology, nanomaterials, nanostructures, vaccine, diagnostics

## Abstract

The COVID-19 pandemic caused by highly-infectious virus namely severe acute respiratory syndrome coronavirus 2 (SARS-CoV-2) has resulted in infection of millions of individuals and deaths across the world. The need of an hour is to find the innovative solution for diagnosis, prevention, and cure of the COVID-19 disease. Nanotechnology is emerging as one of the important tool for the same. In the present review we discuss the applications of nanotechnology-based approaches that are being implemented to speed up the development of diagnostic kits for SARS-CoV-2, development of personal protective equipments, and development of therapeutics of COVID-19 especially the vaccine development.

## 1. Introduction

**Figure 1.**
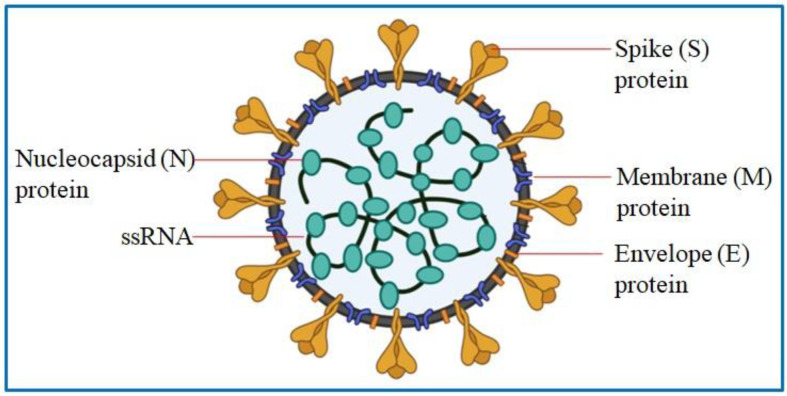
Structure of SARS-CoV-2.

Coronavirus disease 2019 (COVID-19) is an infectious disease caused by virus namely severe acute respiratory syndrome coronavirus 2 (SARS-CoV-2). This virus belongs to Coronaviridae family. SARS-CoV-2 is an enveloped virus with an average diameter of 60–140 nm and contains a single-stranded, positive-sense RNA [1]. The genome of SARS-CoV-2 is around 30 Kb, and it encodes for several accessory proteins and four structural proteins: spike (S), envelope (E), membrane (M), and nucleocapsid (N) (Figure 1) [2].

**Figure 2.**
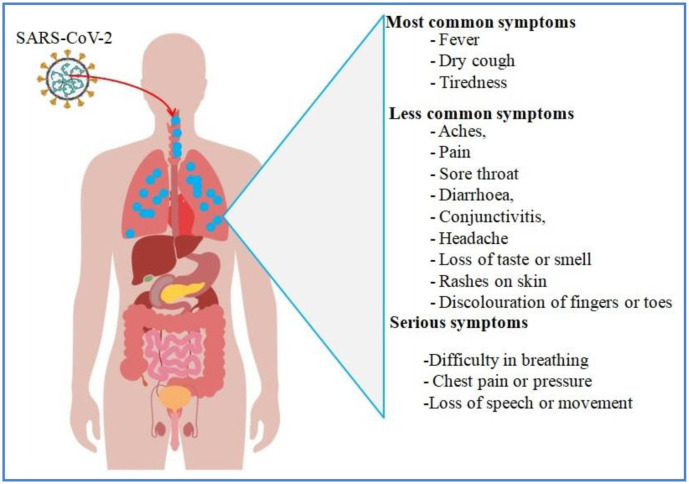
Most common symptoms, less common symptoms and serious symptoms associated with SARS-CoV-2 infection.

The virus, after being first discovered in Wuhan, China, has spread to all over the world and has caused over 1.2 million deaths and over 49.7 million total cases of infection as of 26 July 2021 [3]. Respiratory droplets released during coughing and sneezing and direct contact with infected individual transmit SARS-CoV-2 to healthy individual [4]. The virus mainly affects the respiratory system and can cause acute respiratory distress syndrome and multiple-organ failure, thus leading to patient's death [5]–[6]. COVID-19 affects many organs of the body, so people with COVID-19 may have a wide spectrum of symptoms. Symptoms and signs of the illness may be important to help them and the healthcare staffs they come into contact with know whether they have the disease. Most infected people with SARS-CoV-2 develop mild to moderate illness and recover without hospitalization and specialized treatment. The older people and those with medical complications such as chronic respiratory disease, cardiovascular disease, cancer, and diabetes are more vulnerable and more likely to develop serious illness. Most common symptoms, less common symptoms and serious symptoms associated with SARS-CoV-2 infection are represented in Figure 2.

Mutations in the genome of SARS-CoV-2 have given rise to various new variants reported from many countries namely Brazil, India, South Africa, and the United Kingdom. These new variants are deadlier with higher transmission rate as compared to original strains. Moreover, these variants increase the chance of reinfection and have negative impact on the effectiveness of vaccines-induced immunity [7]. To prevent the spread of the COVID-19, the officials of the countries across the world, enforced various strategies such as suspension of traveling, prolonged lockdown, quarantine, and mandatory facial protection. Such measures indeed decreased the spread of the viral infection, but the limitations of these measures make the unsustainable in a long run due to the involvement of major socioeconomic cost [8]–[10]. Therefore, long-term innovative solution is need of an hour to fight the virus due to limited economic resources. Nanotechnology is one such innovative tool that has emerged with higher success rate in the field of therapeutics and diagnosis and imaging of various diseases [11]. Nanotechnology approaches normally utilizes nanoscale particles (i.e., nanostructures) falling under 1 to 100 nm range. But some authorities extend the definition of nanostructures to include molecules up to 1000 nm size [12]. Nanostructures' small size, large surface to volume ratio, surface charge, bioavailability, biodegradability, biocompatibility, and ability to be modified through surface modification impart nanomaterials unique physicochemical properties that made nanomaterials attractive for biomedical application [13]–[15]. Various types of nanostructures and nanomaterials including metal nanostructures, transition metal oxide nanostructures (emerging nanomaterials that have shown excellence in various fields),carbon-based nanostructures (carbon nanotubes, graphene and graphene oxide), lipid-based nanostructures (nanosized micelles/vesicles),quantum dots, and polymeric nanostructures are being used as theranostic (i.e., therapeutic and diagnostic) agents against viral infections [16]. The last few years have seen increasing use of nanostructures for the treatment of viral infections, such as HIV, hepatitis B virus, Hepatitis C virus, H1N1, herpes simplex virus, human papilloma virus, Zika virus, respiratory syncytial virus, and human norovirus [17]–[19]. During the start of COVID-19 pandemic biomedical researchers proposed that nanotechnology may play an important role in managing the COVID-19pandemic [20]–[24]. The prediction was surely found to be true as can be gauged from the related reports emerging across the globe. For example, scientists have taken advantage of nanotechnology to develop biosensors in order to detect SARS-CoV-2 in patient samples, and to develop antiviral products that can be implemented to prevent the transmission of SARS-CoV-2 [25]. Along with this, nanotechnologies have also been successfully used and are being used to develop therapeutic agents and vaccines against SARS-CoV-2 [25]. In the present review we discuss the nanotechnology-based approaches that are being utilized to mitigate COVID-19 disease. The first section the review summarizes recent studies that have implemented nanotechnology based approaches to speed up the development of diagnostic kits for SARS-CoV-2. The second section discuses the use of nanotechnology based approaches in the development of personal protective equipments such as face masks, sanitizers, surface coatings, etc. in order to prevent the spread of virus. Finally, the third section of the review discuses the application of nanotechnology in therapeutics of COVID-19 especially in the field of vaccine development against SARS-CoV-2.

**Figure 3.**
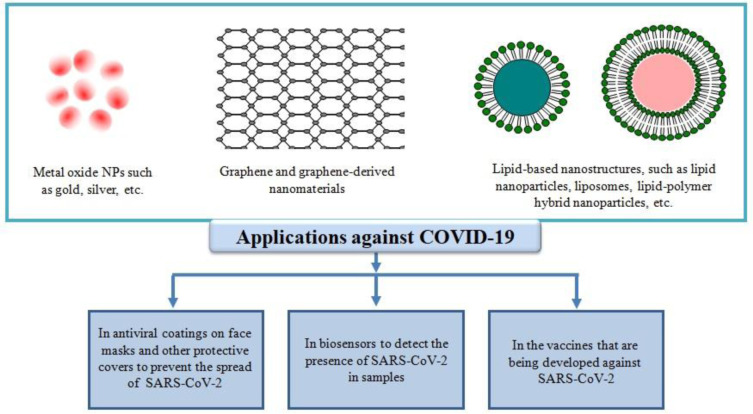
Functional aspects of nanotechnology against COVID-19 pandemic.

## 2. Role of nanotechnology in the detection of SARS-CoV-2

Despite the development of new vaccines against COVID-19 (developed by the companies such as Pfizer, Moderna, Johnson & Johnson, Bharat Biotech International Limited, and Oxford-AstraZeneca), the importance of early rapid detection to control and prevent COVID-19 cannot be further emphasized due to the need to isolate the infected person from rest of the people living in the vicinity to prevent spread of the infection to the still unvaccinated population. There are mainly two types of approaches to detect the presence of SARS-CoV-2. One is the diagnostic approach that involves either detection of viral nucleic acid (i.e. molecular test/assay) or viral antigens (i.e. antigen test). The second one is serological approach that detects the presence of immunoglobulin G (IgG) and/or immunoglobulin M (IgM) or total antibodies produced in a person infected with SARS-CoV-2 [26]. World Health Organization (WHO) has mandated reverse transcription polymerase chain reaction (RT-PCR) as the standard test to diagnose SARS-CoV-2 in patient samples. But RT-PCR is not available everywhere due to economic constraints and the lack of infrastructure, and its results could take from few hours to many days due to insufficient human or lab capacities [27]. This time lag can lead to the spread of COVID-19 to a wider community and loss of precious life that could have been saved only if the person had been diagnosed earlier. In these circumstances, point-of-care testing that should be reliable, cheap, and fast and should provide results within short time window has become need of an hour for the prevention of COVID-19 pandemic. Because nanostructures are of similar size to that of SARS-CoV-2, nanostructures can bind to the virus and thus can be utilized for therapy and detection of COVID-19 infection [28]. The use of nanotechnology and nanostructures in order to develop diagnostic and serological tests for COVID-19 infection is increasing greatly [29]–[32]. Nanomaterials can be functionalized with biomolecules to detect and amplify signal in colorimetric detection of pathogens [33], and large surface area of nanostructures increases capture efficiency of, and thus sensitivity of a biosensor [34]. Similarly, the functionalization of nanostructures with antibodies against SARS-CoV-2 increases sensitivity and affinity of biosensor for the analyte and enhances signals (e.g. electrochemical, electrochemiluminescent, magnetic, or optical signals) thus making the detection of target molecule and of COVID-19 infection easier [35]. Table 1 lists the examples of nanomaterials that are being used to devise biosensor to detect SARS-CoV-2.

Many biosensing techniques for the detection of SARS-CoV-2 have utilized gold nanostructures (Table 1). Because these nanostructures are easier to synthesize, biocompatible, and physicochemically stable [36]. Karami and coworkers [37] devised a colorimetric detection technique for SARS-CoV-2 RNA by attaching spherical nucleic acids to the surface of gold nanostructures. The biosensing system included a 4-nucleotide palindromic linker to probe the E gene region of SARS-CoV-2 RNA. The assay was comparable in sensitivity to the real-time RT-PCR.

In an interesting approach, Sil and group [38] designed a type of qualitative immunoassay named as rapid flow-through dot-blot immunoassay. The group used gold nanostructures functionalized with antibodies to detect SARS-CoV-2 specific IgG in human serum, with sensitivity and specificity of 98.8% and 98%, respectively.

Ventura et al. [39] constructed gold nanostructures-based biosensor to detect SARS-CoV-2 in nasal and throat swabs. The researchers prepared three types of gold nanostructures, each functionalized with antibodies against spike, envelope, or membrane surface proteins of SARS-CoV-2. The three types of gold nanostructures were mixed in equal (1:1:1) ratio to make a colloidal solution, when interacted with virus particles, gave red color, which could be measured at 560 nm to quantify viral load. The detection method is a faster and better alternative to the RT-PCR method, because it does not require preparatory steps, such as RNA extraction and amplification. Further, the detection technique can quantify the viral load and severity of the viral infection, because the technique detects viral particles rather than SARS-CoV-2 RNA.

Moitra et al. [40] also utilized gold nanostructures and developed a colorimetric assay to detect SARS-CoV-2 in patient samples. The researchers functionalized gold nanostructures with thiol-modified antisense oligonucleotides specific for N-gene of SARS-CoV-2. In the presence of target RNA sequence of SARS-CoV-2, the functionalized gold nanostructures agglomerated, which could be detected by UV-Visible absorbance spectroscopy and other techniques including transmission electron microscopy, and hyper spectral microscopy. Further, when RNA samples with functionalized gold nanostructures were treated with enzyme RNase H at 65 °C for 5 min, the samples were precipitated that could easily be seen by naked-eye, thus avoiding the need for any sophisticated instrument to detect SARS-CoV-2. The bioassay was selective for SARS-CoV-2, because it did not produce any distinct change in absorbance in the presence of Middle East respiratory syndrome coronavirus (MERS-CoV) RNA; the limit of detection was 0.8 ng µL^−1^ for SARS-CoV-2.

Gold, silver and other nanostructures can show plasmonic effects that when combined with surface-enhanced Raman scattering (SERS) spectroscopy or surface-enhanced infrared absorption (SEIRA) spectroscopy can be used to detect small analyte at a very low concentration [41] and in the construction of point of care devices for virus detection [42],[43]. Jadhav and coworkers [44] exploited surface-enhanced Raman spectroscopy (SERS) by coupling it with a microfluidic platform that contained silver or gold nanostructures coated carbon nanotubes micro channels. Silver and gold nanostructures acted as plasmonic materials that could increase Raman intensity of even traces of SARS-CoV-2 in samples (eyes, nasal, throat, and saliva swabs) and helped in rapid of detection of the virus in the sample [44]. In another surface-enhanced Raman spectroscopy based lateral-flow immunoassay, Liu et al. [45] showed that using complete Ag shell on SiO_2_ core (i.e. core-shell nanostructures) resulted in 800 times increase in the sensitivity of biosensor for the detection of anti-SARS-CoV-2 IgM/IgG in comparison with that of standard gold nanostructures-based lateral-flow immunoassay method.

Recently, Qiu and coworkers [46] developed a dual-functional ultrasensitive biosensor based on plasmonic effects (plasmonic photo thermal effect and localized surface plasmon resonance) to detect SARS-CoV-2, with a detection limit of 0.22 × 10^−12^ M. The biosensor included two-dimensional gold nanoislands, a type of nanostructure, functionalized with complementary DNA, which when hybridized to RNA of SARS-CoV-2 could detect the presence of the virus.

Zhu et al. [47] devised a new diagnostic assay based on reverse transcription loop-mediated isothermal amplification method in combination with a nanostructure-based lateral flow biosensor to diagnose COVID-19 in patients. Zhu's group reported 100% sensitivity for the diagnostic assay, after analyzing 33 oropharynx swab samples from COVID-19 patients. The assay also showed very high specificity of 100% for COVID-19 negative samples. The assay was very fast and took only an hour to complete.

Wang's group [48] have recently exploited modified selenium nanostructures to prepare a lateral flow immunoassay kit that can detect anti-SARS-CoV-2 antibodies (IgG and IgM) in human serum, with naked-eye in only 10 minutes. The researchers tested the bioassay in 90 COVID-19-diagnosed patients and 263 non-infected controls and reported 93.33% sensitivity and 97.34% specificity.

Cobalt-functionalized TiO_2_ nanotubes (Co-TNTs) was used by Vadlamani et al. [49] to synthesize a non-expensive and highly sensitive electrochemical sensor that can detect the receptor-binding domain (RBD) domain of spike protein of SARS-CoV-2 in the concentration range of 14 to 1400 nM.

In the place of commonly used gold nanostructures in biosensor to detect SARS-CoV-2, some research groups used carbon nanotubes [41],[48], graphene [49],[50], and graphene-based (graphene oxide or reduced graphene oxide) [51] nanomaterials due to carbon nanotube's and graphene's high conductivity and biocompatibility [52],[53], and benefit of lower cost [29]. Further, the modification of graphene's surface with functional groups imparts the biosensor with very high sensitivity and ability to recognize biological molecule [54]. For example, Seo and colleagues [49] developed a graphene field-effect transistor (FET)-based biosensor device for the detection of SARS-CoV-2 in clinical samples. In this sensor, the researchers coated graphene sheets of field-effect transistor with a specific antibody against SARS-CoV-2 spike protein. The biosensor could detect the SARS-CoV-2 spike protein at concentration as low as of 1 fg/mL in phosphate-buffered saline (PBS) and of 100 fg/mL in clinical transport medium. The biosensor could further detect SARS-CoV-2 in clinical samples with the limit of detection of 2.42 × 10^2^ copies/ml. The sensor can discriminate between SARS-CoV-2 antigen protein and MERS-CoV antigen protein, thus restricting any chance of cross-reactivity with MERS-CoV [49]. Li and collaborators [50] achieved amplification-free detection of SARS-CoV-2 RNA via a biosensor that included graphene field-effect transistor decorated with morpholino-modified gold nanostructure. The tests showed very low limit of detection of SARS-CoV-2 RNA in phosphate buffer saline (0.37 fM), throat swab (2.29 fM), and serum (3.99 fM); and the test took only 2 minutes to ascertain the SARS-CoV-2 infection in clinical throat swabs sample [50].

In another study, Huang et al. [55] developed lateral-flow assay based on colloidal gold nanostructures to detect IgM antibody against SARS-CoV-2 in serum samples of patients through immunochromatography. This assay can give results in only 15 minutes, and it needs only 20 µL of serum sample for each test in comparison with RT-PCR test that needs around 100 µL of the sample. The sensitivity and specificity of the nanostructures based lateral-flow assay were 100 and 93.3% respectively.

Similarly, Mertens and group [56] have prepared a diagnostic strip named as Ag Respi-Strip, which is based on the principles of immunochromatographic assay. The diagnostic strip utilized anti-SARS-CoV-2 (nucleocapsid protein) antibodies conjugated with colloidal gold nanostructures. The sensitivity and specificity of the test were 57.6% and 99.5%, respectively.

Wen et al. [57] reported a rapid point-of-care (POC) lateral-flow immunoassay based on colloidal gold nanostructures to diagnose SARS-CoV-2. The researcher's functionalized gold nanostructures to anti–human IgG, sprayed it onto the pre-treated conjugate pad that already contained SARS-CoV-2 nucleocapsid protein, and employed a change in color as a marker for the presence of viral protein in patient samples. The sensitivity and specificity of the assay were 69.1% and 100%, respectively.

Shan and coworkers [58] developed a novel biosensor that detects SARS-CoV-2 from exhaled breath. The sensor is based on the principle that during viral infection cells produce—due to interaction between host cells and virus—volatile organic compounds, which are present in the exhaled breath of a patient and thus can be used as a biomarker for the detection of a disease [59]. The sensor constructed by Shan's group (2020) [60] included a sensing layer composed of organic ligands and gold nanostructures. When exposed to volatile organic compounds present in exhaled breath, the sensing layer shrinks or swells and causes a volume change in the nanomaterial film that was captured by change in electric resistance. Although this technique needs to be validated in a larger cohort size, it can still be used for the rapid screening of COVID-19.

Fabiani et al. [54] have developed an electrochemical immunoassay for rapid detection of SARS-CoV-2 in saliva samples. Screen-printed electrodes used in the assay were based on carbon black nanomaterial, which due to its cost-effectiveness, electro catalytic properties, and biocompatibility is gaining popularity in the designing of electrochemical biosensors [61]. The assay could detect spike protein or nucleocapsid protein of the SARS-CoV-2. A detection limit of 100 fg/ml was found for nasopharyngeal swab samples from COVID-19 patients.

In a very interesting approach, Vaquer et al. [62] devised a non-invasive method for detecting SARS-CoV-2 proteins trapped in surgical face masks. The researchers used antibody-decorated gold nanostructures, which when comes in contact with SARS-CoV-2 antigens trapped in surgical face masks produce colorimetric signal; the whole assay takes only less than 10 minutes to complete.

**Table 1.** Some recent studies using nanomaterials in the biosensor for detecting SARS-CoV-2.

Nanomaterial/nanostructures	Detection of molecule	Limit of detection	Study
Gold nanostructures	Spike protein	5 µg·mL^–1^	[63]
Gold nanostructures	Spike protein	80 copies mL^−1^ in contaminated water	[64]
Gold nanostructures	anti-SARS-CoV-2 IgG	Semi-quantitative	[37]
Gold nanostructures	RNA	4 copies/µL	[65]
Gold nanostructures	N gene and E gene	300 copies/µL of E gene; 225 copies/µL of N gene	[66]
Gold nanostructures	Viral proteins of SARS-CoV-2 trapped in surgical face masks	3 ng mL^−1^	[62]
Gold nanostructures	RNA	50 RNA copies per reaction	[67]
Gold nanostructure	16S rRNAs; N gene	(Not Reported)	[68]
Gold nanostructures	anti-SARS-CoV-2 IgG antibodies	3.2 nM	[69]
Gold nanostructures	N gene	0.18 ng/µL	[39]
Gold nanostructures	Viral particles	Viral loads corresponding to C_t_ = 36.5	[38]
Gold nanostructures	RNA	10 copies/µL	[70]
Gold nanostructures	anti-SARS-CoV-2 IgM	(Not reported)	[55]
Gold nanostructures	Highly conserved nucleoprotein	250 pg/mL	[56]
Gold nanostructures	anti-SARS-CoV-2 IgG	(Not reported)	[57]
Gold nanostructures	Volatile organic compounds from breath	(Not reported)	[58]
Gold Nano star	Spike protein and the virus	130 fg/mL for antigen and 8 particles/mL for virus	[71]
Gold nanoislands	RNA	0.22 pM	[44]
Gold nanoisland films	RNA	2.98 copies µL^−1^	[72]
Gold nanostructures and spherical nucleic acid	RNA	(Not reported)	[73]
Gold nanostructures and spherical nucleic acid	RNA	Six copies of ssDNA per reaction	[36]
Gold nanostructures and graphite	Spike protein	229 fg mL^−1^	[74]
Gold and silver nanostructures	Spike protein	0.77 fg mL^−1^ in PBS; 6.07 fg mL^−1^ in saliva; 7.60 fg mL^−1^ in serum; and 0.10 pg mL^−1^ in blood	[75]
(Not Reported)	SARS-CoV-2 total antibody	(Not Reported)	[76]
Gold-platinum core-shell nanostructures	Subunit S1 of spike protein (S)	11 ng mL^−1^	[77]
Gold nanostructure (AuNP)-decorated graphene field-effect transistor (G-FET) sensor	RNA	0.37 fM in PBS; 2.29 fM in throat swab; and 3.99 fM in serum	[50]
Super paramagnetic nanostructures	anti-SARS-CoV-2 IgM and IgG	10 ng/mL for IgM and 5 ng/mL for IgG	[78]
Lanthanide-doped nanostructures	anti-SARS-CoV-2 IgG	(Not Reported)	[79]
Selenium nanostructures	anti-SARS-CoV-2 IgM and IgG	20 ng/mL for IgM and 5 ng/mL for IgG	[46]
Au/Ag coated carbon nanotubes	Live virus	(Not Reported)	[41]
Europium-chelate-based fluorescent nanostructures	RNA	1,000 TU (transduction units) mL^−1^	[80]
Ag shell on SiO_2_ core (SiO_2_@Ag)	anti-SARS-CoV-2 IgM/IgG	1 pg/mL	[43]
(Not Reported)	Total antibody (IgA, IgM, and IgG) against SARS-CoV-2	(Not Reported)	[81]
Cobalt functionalized-TiO_2_ nanotube	RBD domain of S protein	~0.7 nM	[47]
Carbon black	Spike (S) protein or Nucleocapsid (N) protein	19 ng/mL for S protein; 8 ng/mL for N protein	[60]
Carbon nanotubes	RNA	6.4 copies/µL in PBS and 9.2 copies/µL in 50% human saliva	[48]
Graphene	Spike protein	1.6 × 10^1^ pfu/mL in culture medium; 2.42 × 10^2^ copies/mL in clinical samples; 1 fg/mL in phosphate-buffered saline; 100 fg/mL in clinical transport medium	[49]
Graphene	Spike protein	∼3.75 and ∼1 fg/mL in artificial saliva and phosphate-buffered saline, respectively	[82]
Reduced-graphene-oxide Nano flakes	Antibodies to spike S1 protein and its receptor-binding-domain (RBD)	2.8 × 10^−15^ M for spike S1 protein and 16.9 × 10^−15^ M receptor-binding-domain	[51]
Quantum dot	Antibodies	17.5 pM for nucleocapsid antibody and 24.4 pM for S1 antibody	[83]
Quantum dot Nano beads	SARS-CoV-2 total antibody	(Not Reported)	[84]
Enzyme-DNA hybrid Nano complexes	RNA	~8 RNA copies/µL	[85]
Streptavidin coated polymer nanostructures	ORF1ab and N gene	12 copies per reaction	[45]
Aggregation-induced emission (AIE) dye-loaded nanostructure	anti-SARS-CoV-2 IgM and IgG	0.236 mL^–1^ for IgM; 0.125 µg mL^–1^ for IgG	[86]

## 3. Role of nanotechnology in preventing transmission of SARS-CoV-2

To prevent spread of COVID-19, social distancing, mask wearing, and hand sanitizing are being implemented. Similarly, products, such as face masks, face shields, disinfectants, and sanitizer are being used to minimize the spread of SARS-CoV-2 infection in the community. Although face masks protect the person from catching the virus, they do have certain limitations: (1) medical masks recommended for health workers are for single-use only; (2) face masks act only as a barrier to virus transmission, they do not inactivate virus; (3) N95 face masks provide only 95% filtration efficiency for particles more than 0.3 micron size [87], thus particles that are less than 200 nm, such as SARS-CoV-2 can pass through them. Nanotechnology-based solutions are being utilized to improve upon such limitations, because nanostructures can infiltrate viral coronae, can bind to the virus and prevent its attachment to the host cell surface during entry, and can inhibit viral replication thus preventing spread of infection [88],[89]. The embedding of nanomaterials can provide antiviral properties to (i.e., viral inactivation capability) and improve filtration ability of protective clothing [90]. Further, nanostructures can persist for longer duration and can be effective at a low concentration, which makes their use in disinfectants to sterilize air, physical surfaces, or skin beneficial [91]. Nanostructures can prevent spread of virus by various mechanisms: They can interact with the viral glycoproteins, thus prevent binding and penetration of viral glycoproteins to the host cell [91]; and on exposure to UV radiation, the nanostructures can generate reactive oxygen species that oxidize the viral membrane or envelope [91],[92]. Table 2 has listed some nanotechnology-based products that have claimed to prevent SARS-COV-2 transmission in community. The products are using various nanomaterials, including graphene, copper oxide, titanium dioxide, and silica, nanene (a type of graphene) in products, such as face masks, face shields, fabrics, packaging material, air filters, and coating (Table 2).

**Table 2.** Products based on nanotechnology to prevent SARS-CoV-2 transmission.

Products	Nanostructures	Company/Organization	Country
Graphene Mask	Graphene	Flextrapower Inc	USA
Nano shield	CuO	Nanoveu Inc	Australia
Virucidal Graphene-Based Composite Ink	Graphene and Ag	ZEN Graphene Solutions Ltd	Canada
Guardian G-Volt respiratory mask	Graphene	LIGC Applications Ltd	USA
Co-Mask	Graphene	Directa Plus PLC	UK
Antiviral fabrics	Cu	Promethean Particles Ltd	UK
MVX Nano MaskTM	TiO_2_	MVX Prime Ltd	UK
Nano Silver Sanitizer	Ag	Shepros Sdn. Bhd.	Malaysia
ReSpimask® VK (Virus Killer)	CuO	Respilon Group S. R. O.	Czech Republic
Diamond Face Mask	Diamond	Master Dynamic Limited	China
NANOHACK	CuO	Copper 3D Antibacterial Innovations	Chile
G1 Wonder Face Masks	Graphene	Nanomatrix Materials	India
Nanocoating	(Not reported)	Nanoksi Finland	Finland
Nanofense	Ag	Applied Nanoscience Inc.	USA
Transparent stretchable PVC film for use in packaging	Silver and silica	Alpes and Nanox	Brazil
Graphene Face Mask	Graphene	Medicevo	USA
Nanofiber Membranes	(Not reported)	BYU's College of Engineering with Nanos Foundation	USA
ZEN's Virucidal Ink	Graphene	ZEN Graphene Solutions Ltd.	Canada
Nano-coated air filters	(Not reported)	University of Houstan	USA
Graphene Enhanced Protective Face Mask	Nanene	Versarien	UK
Nano Textile Coating	(Not reported)	IIT Madras and Muse	India
Rubber, paint, coating, and cosmetics	ZnONPs	Brüggemann	Germany

**Source:** Nanotechnology Products Database (2021) [93]; Nanotechnology in battle against coronavirus (n.d.) [94].

The nanostructures impregnated to the face masks can kill viral particles remained in the masks without altering filtering properties of face masks and thus can ensure further reduction in risk of viral infection due to incorrect handling and disposal of face masks [95]. Very recently, a research team from Korea Advanced Institute of Science and Technology (KAIST), Daejeon, South Korea has developed a nanotechnology-based filter that retains excellent filtering efficiency even after washing for more than 20 times [96]. The use of such filters can solve the problem of shortage of face masks. The researchers used orthogonal nanofibers and insulation block electrospinning process to create the face mask. According to the scientist, the nanofiber filter is water-resistant and shows 94% filtration efficiency after 20 repeated bactericidal tests with ethanol [96].

Similarly, Queensland University of Technology-based research team has constructed biodegradable face masks based on nanotechnology [97]. The face mask is made of cellulose nanofiber obtained from plant waste and agricultural waste. The breathable nanocellulose material can filter particles smaller than 100 nm, which is the size of several human viruses, including SARS-CoV-2.

Wakamono, which is a Vietnam based company working in field of nanotechnology, has announced that it has created the world's first anti-coronavirus surgical masks [98]. The mask is made of GECIDE fabric technology that utilizes organic nanostructures and can inactivate SARS-CoV-2 up to 99%. The mask is also effective against influenza A H1N1 and poliovirus I [98].

Tremiliosi et al. [99], in a recent study, functionalized polycotton fiber with silver nanostructures by the pad-dry-cure method. The composite fibers were found to inhibit the SARS-CoV-2 only after two minutes incubation. The fiber has the potential to be used in textile materials that can stop the transmission of SARS-CoV-2.

Jeremiah and group [100] have recently showed the antiviral effect of silver nanostructures on SARS-CoV-2 viral suspension. This study showed that silver nanostructures can be used as surface coating to deactivate SARS-CoV-2 and prevent their further transmission. The angiotensin-converting enzyme 2 (ACE2) receptor-expressed by various organs, such as liver, heart, kidney, lungs, etc. [101] plays an important role in the entry of SARS-CoV-2 into host cells [102]. But, the receptor can also play protective role in acute lung injury [103]; this fact prompted Aydemir and Ulusu [104] to propose that nanomaterials coated with ACE2 can be used to prepare clothes, mask, gloves, and other protective covers to sequester virus and block their entry into the host.

Products based on graphene and its derivatives (graphene oxide, reduced graphene oxide, and graphene quantum dots) as argued by Raghav and Mohanty [105], have the potential to be used against SARS-CoV-2 infection. The mono- or multi-layers graphene and its derivatives can be applied either as coating on fabrics to keep them dry to prevent aerosol transmission of SARS-CoV-2, or as a mist spray and cleaning solution to sanitize the infected surfaces of an object or human body [105]. The antiviral effect of graphene oxide and its Nano composite on feline coronavirus, an enveloped virus, has shown by Chen et al. [106]. Similar application of graphene oxide against SARS-CoV-2, also an enveloped virus, can be tried by using coatings of graphene oxide on clothes to make antiviral protective products.

De Maio and group [107] showed that polyurethane and cotton both used in personal protective equipment when functionalized with graphene nano platelets or graphene oxide can entrap SARS-CoV-2. When the SARS-CoV-2 culture was incubated with or filtered through the functionalized polyurethane and cotton material, the capacity of the virus to infect Vero cells (African green monkey kidney epithelial cell) decreased. However, the concentration range of graphene oxide (0.06–0.5 mg/mL) used in the experiment is not realistic for the purposes of therapeutics against SARS-CoV-2. Unal's group [112] in different experiment via molecular docking method and in vitro method showed that graphene oxide sheets could decrease infectivity of SARS-CoV-2 by interacting with its viral spike protein. The efficacy of graphene oxide sheets were also shown to be persisted when different mutations were present in the viral spike protein [112].

The Guardian G-Volt Respiratory mask by LIGC Applications employs a graphene filtration system [109]. This mask is 99% effective against particles over 0.3 micrometers, in comparison with N95 respirator mask that can only blocks 95 per cent of particles over 0.3 micrometers, thus having the potential to prevent SARS-CoV-2 transmission [113].

Coating of silver nanocluster/silica composite onto disposables facial FFP3 masks (3M™) have been shown to reduce the titer of SARS-CoV-2 to zero [114]. The application of the antiviral coating can be extended to other surfaces, such as ceramic, glass, and metallic to prevent the spread of the virus in public areas [115].

Kumar's group [96] has created a photoactive antiviral mask, which was coated with hybrid of copper nanostructures and shellac, a hydrophobic biopolymer. As per researchers, under the sunlight the temperature of the mask could reach >70 °C, which in turn give rise to free radicals that can disrupt the membrane of virus-like particles and give the mask self-sterilization capability. The mask can be a great asset against protection from SARS-CoV-2 and COVID-19 pandemic.

Although the COVID-19 pandemic is witnessing the emergence of many commercial products (e.g., face masks, coatings, etc.) that claim to be using nanostructures and be effective against SARS-CoV-2, such claims must be duly scrutinized to save consumer from any harm from potential exposure to the virus. For example, Blevens et al. [112] evaluated the claims of 40 cloth face masks that claimed to be using either silver or silver nanostructures and to have antiviral properties. The researchers concluded that the claims made by companies on using nanostructures in face masks are not always true, and further the filtration efficiency might not up to the standard certification requirement. The researchers proposed stricter regulation of products by governmental agencies to ensure efficacy of the face masks for safety of the consumer [112]. Similarly, there is a persisting concern among scientist due to the long-term effect of nanomaterial application in protective fabrics to the environment and to the person wearing the protective covering [86]. Further, the widespread uses of masks have inevitably accelerated the release of nanoplastics, micro plastics, and chemical pollutants from disposable masks to the environment [113],[114]. If the scientists could combine use of nanomaterials in masks with new nanotechnology that degrade micro plastics such as that shown by Kang's group [115], then it would be better for the environment and for people's health. Another approach to decrease the toxicity of metal nanostructures to the person using protective fabrics as suggested by Sportelli et al. [123] in a review article was to impregnate copper salt along with nanomaterials into the masks, personal protective clothing, and fabrics.

## 4. Role of nanotechnology in therapeutics development against SARS-CoV-2

Multiple research groups all over the world are working on therapeutic agents to prevent COVID-19. For example, as of 31 July 2021, more than 200 vaccines (including both in clinical and preclinical evaluation) against SARS-CoV-2 are in development [116]. Among these, seven (BNT162b2 or Comirnaty by Pfizer–BioNTech, mRNA-1273 by Moderna, AZD1222 by Oxford–AstraZeneca, Ad26.COV2.S by Janssen, Covishield by Serum Institute of India, BBIBP-CorV by Sinopharm-BBIBP, and CoronaVac by Sinovac) have been recognized by the World Health Organization for emergency use [117]. Recently, the U.S. Food and Drug Administration (FDA) has approved the Comirnaty. It is a vaccine for preventing coronavirus disease 2019 (COVID-19) in people aged 12 years and older. Comirnaty contains a molecule called messenger RNA (mRNA) with instructions for producing a protein from SARS-CoV-2, the virus that causes COVID-19. Comirnaty does not contain the virus itself and cannot cause COVID-19.Before the advent of any officially approved vaccine for use against COVID-19, the first approach of the scientists all over the world was to repurpose existing medicines including chloroquine, hydroxychloroquine, favipiravir, interferon, lopinavir, nitazoxanide, remdesivir, ribavirin, ritonavir, and umifenovir [118]. But interim results of the World Health Organization's SOLIDARITY trial found no significant effect for such drugs on COVID-19 patients [119]. In these circumstances, vaccines and other therapeutics agent that are being developed are only hope for protecting people from SARS-CoV-2. Many vaccines that are in pipeline against COVID-19 are exploiting nanotechnology, because nanostructures can increase the solubility of drugs, cross blood–brain barrier, increase half-life of drugs, decrease toxicity of drugs, and in some cases can deliver drug to the target organ and tissue inside the body, thus decreasing the toxicity of drugs [120]–[122]. Nanomaterials such as liposomes, micelles, polymeric nanostructures, lipid nanostructures (LNPs), and lipid-polymer hybrid nanostructures have also shown their importance for drug delivery [123]. Further, the surfaces of nanostructures can be modified by various molecule and functional group for desired biological properties.

Virus-like particles (VLPs) are a type of lipid-polymer hybrid nanostructures that are derived from self-assembly of viral capsid or envelope proteins and are non-infectious because they do not contain genetic material and ability to replicate [124],[125]. VLPs mimic virus particles and can be constructed via recombinant technology to present peptide antigen that could elicit immune response, and thus can be used in the development of vaccines [126]. The VLPs can be engineered to display antigen or epitope in highly ordered repeating manner, which gives VLPs high immunogenicity that can generate strong cellular and humoral immune responses with induction of high affinity antibodies [127],[128]. Further, VLPs due to its small size range (20–200 nm) [129] can also act as an efficient nanoacarrier for foreign (i.e. not related to VLPs itself) antigens that helps them in readily taken up by antigen presenting cells to stimulate the immune system [130],[131].

VLP-based vaccines have been successfully developed against many inflammatory or infectious diseases, such as human papillomavirus, hepatitis B virus, etc. [132]. Many companies are developing VLPs-based vaccines (some are in clinical phase, while others are in preclinical phase) against SARS-CoV-2 (Table 3).

Medicago Inc, a Canadian company, has utilized plant-derived VLPs with vaccine adjuvants from GSK and Dynavax to develop a COVID-19 vaccine [116]. The CoVLP technology utilizes *Nicotiana benthamiana* plant as a bioreactor for the vaccine production by using *Agrobacterium tumefaciens* as disarmed vector that transfer episomal DNA containing SARS CoV-2 spike protein gene to the nucleus of the plant cells [133]. Similarly, ARTES Biotechnology is using two VLP-based technologies—enveloped VLP technology called METAVAX and capsid VLP technology called SplitCore—for the development of COVID-19 vaccines [134]. VBI Vaccines Inc. is also making COVID-19 vaccine named VBI-2902a using enveloped VLPs as platform, results of which recently have been published by Fluckiger and collaborators [135]. The researcher showed preliminary efficacy of the vaccine against SARS-CoV-2 in Syrian golden hamsters by suppressing clinical disease and lung inflammation [135].

**Table 3.** Virus-like particles (VLPs) Vaccine against SARS-CoV-2.

VLPs-based COVID-19 vaccine in clinical development		
Vaccine	Developer	Trial phase
CoVLP	Medicago; GSK; Dynavax	Phase 3
SARS-CoV-2 VLP Vaccine	The Scientific and Technological Research Council of Turkey; Dr Abdurrahman Yurtaslan Ankara Oncology Training and Research Hospital; MonitorCRO; Nobel Pharmaceuticals	Phase 2
IVX-411	Icosavax, Inc.; Bill & Melinda Gates Foundation; Amgen; Seqirus	Phase 1/2
VBI-2902a	VBI Vaccines Inc.	Phase 1/2
ABNCoV2	ExpreS2ion Biotech; Bavarian Nordic A/S	Phase 1/2

VLPs-based COVID-19 vaccine in preclinical phase		
Vaccine	Developer	Country

VLP	Max Planck Institute for Dynamics of Complex Technical Systems	Germany
Virus-like particle-based Dendritic Cell(DC)-targeting vaccine	University of Manitoba	Canada
VLP	Bezmialem Vakif University	Turkey
Enveloped Virus-Like Particle (eVLP)	VBI Vaccines Inc.	USA
S protein integrated in HIV VLPs	IrsiCaixa AIDS Research/IRTA-CReSA/Barcelona Supercomputing Centre/Grifols	Spain
VLP + Adjuvant	Mahidol University/ The Government Pharmaceutical Organization (GPO)/Siriraj Hospital	Thailand
Virus-like particles, lentivirus and baculovirus vehicles	Navarrabiomed, Oncoimmunology group	Spain
Virus-like particle, based on RBD displayed on virus-like particles	Saiba GmbH	Switzerland
ADDomer™ multiepitope display	Imophoron Ltd and Bristol University's Max Planck Centre	UK
VLP	OSIVAX	France
eVLP (enveloped VLP)	ARTES Biotechnology	Germany
VLPs-based COVID-19 vaccine in preclinical phase
Vaccine	Developer	Country
VLPs peptides/whole virus	University of Sao Paulo	Brazil
VLPs produced in baculovirus expression vector system or BEVS	Tampere University	Finland
Plant derived VLP	Shiraz University	Iran
Myxoma virus co-expressing S, M, N and E proteins	Arizona State University	USA
Plasmid driven production of VLPs containing S, M, N and E proteins of SARS-CoV-2	Arizona State University	USA
Virus Like Particle with RCB	Berna Biotech Pharma	Switzerland
RBD-HBsAg VLPs or Receptor Binding Domain SARS-CoV-2 Hepatitis B surface antigen VLP Vaccine	SpyBiotech/Serum Institute of India	India
MVA encoded VLP	GeoVax/BravoVax	China
Drosophila S2 insect cell expression system VLPs	ExpreS2ion	Denmark
VLP-recombinant protein with adjuvant	Osaka University/ BIKEN/ National Institutes of Biomedical Innovation	Japan
Lipid nanostructures (LNP)-encapsulated mRNA cocktail encoding VLP	Fudan University/Shanghai JiaoTong University/RNACure Biopharma	China

**Source:** World Health Organization [116]; COVID-19 treatments and vaccine tracker [136]

Lipid nanostructures (LNPs) are another nanotechnology-based platform that is being used to develop vaccines against SARS-Co-2. Most of the mRNA-based COVID-19 vaccines are using lipid nanostructures to encapsulate mRNA for delivery into cells. Lipid nanostructures protect mRNA against enzymatic degradation, help mRNA in escaping from endosomes and allow release of the enclosed mRNA into the cytosol, and improve the cellular uptake and thus expression of mRNA by many-fold compared to naked mRNA inside the host cells [137]–[139]. Among the seven approved vaccine for COVID-19 by the World Health Organization [117], two are mRNA based and use lipid nanostructures as a nanocarrier for the delivery: Comirnaty (BNT162b2) by Pfizer, BioNTech and Fosun Pharma; and Moderna COVID-19 Vaccine (mRNA-1273) by Moderna, Biomedical Advanced Research and Development Authority (BARDA), and National Institute of Allergic and Infectious Diseases (NIAID). Along with these two, many other mRNA-based vaccines being developed against SARS-CoV-2 are using LNPs: ARCoV, CVnCoV, DS-5670a, MRT5500, PTX-COVID19-B, and ChulaCov19 (Table 4).

Pfizer, in collaboration with BioNTech, has developed the dual-dose vaccine Comirnaty^®^BNT162b2 against SARS-CoV-2 [140]. In Comirnaty^®^, nucleoside-modified mRNA—that encodes receptor binding domain (RBD) of the SARS-CoV-2 spike protein—has been encapsulated inside lipid nanostructures. In phase I/II trials in the USA and in Germany, the vaccine was found to induce robust immunogenic responses [140]. The UK was the first and Canada was second country to approve emergency use of Comirnaty against SARS-CoV-2. To see the efficacy of the vaccine among different population groups such as pregnant women over the age of 18, children under 12 years old, and adolescents between the age 12 and 15, the clinical trials have been started in different countries [140]. With an efficacy of 95%, Comirnaty^®^ has been approved in 85 countries as of 16 May 2021 [141].

Similarly, Moderna has developed mRNA-1273 vaccine based on lipid nanostructures against SARS-CoV-2. The mRNA-1272 vaccine is also two-dosed like as that of Comirnaty^®^ of Pfizer. The vaccine exploits lipid nanostructures to encapsulate nucleoside-modified mRNA that encodes SARS-CoV-2 spike glycoprotein stabilized in its prefusion conformation [142]. In the preliminary studies done in mice [142] and primates [143], the vaccine has been found to protect against SARS-CoV-2 infection. In The Coronavirus Efficacy (COVE) phase 3 randomized, observer-blinded, placebo-controlled, multicenter trial among 30,420 volunteers in the USA, the mRNA-1273 was found to be safe and efficacious for preventing SARS-CoV-2 infection [144]. The Moderna mRNA-1273 vaccine has an efficacy of 94.1% and has been approved for use in 46 countries as of 16 May 2021 [141].

The other example of COVID-19 vaccine that is using nanostructures platform is NVX-CoV2373 developed by Novavax. It is a recombinant nanostructure vaccine that displays trimeric full-length SARS-CoV-2 spike glycoproteins [145]. Novavax is using its own saponin-based Matrix-MTM adjuvant technology in the clinical trial for the NVX-CoV2373 vaccine [145]. In the phase 3 randomized, observer-blinded, placebo-controlled, multicenter trial in the UK, the two-dosed regimen of NVX-CoV2373 gave an efficacy of 89.7% against SARS-CoV-2 infection and also found to be highly effective against the B.1.1.7 variant [146]. In a recently published research articles, the combination of NVX-CoV2373 and recombinant hemagglutinin (HA) quadrivalent nanostructure influenza vaccine (qNIV) protected hamsters challenged with SARS-CoV-2 [147]. The combination vaccine can also protect against seasons influenza. He et al. [148] have reported a next generation vaccine strategy against SARS-CoV-2 using self-assembling protein nanostructures (SApNPs) technology. The researchers displayed receptor binding domain (RBD) and SARS-CoV-2 spike as vaccine antigens on SApNPs. The nanoparticle system elicited a potent immune response by T-cells in mouse model [148].

In a proof-of-concept study, Zeng's group [149] engineered endogenous untranslated regions (UTRs) of mRNAs termed as NASAR to enhance SARS-CoV-2 antigen production. The engineered mRNA when delivered using lipid-derived TT3 nanostructures induced improved antibody response in mice in comparison with formulation based on FDA-approved lipid nanostructure delivery system. The researchers argued for the further development of NASAR system into alternative COVID-19 vaccines.

Various research groups have utilized nanostructures such as liposomes, reconstituted lipoproteins, and cell-membrane-derived nanostructures that mimick cell membrane to counter SARS-Cov-2 infection. These are called nanodecoy or decoy nanostructures. Rao et al. [150] have engineered decoy nanostructures to counter SARS-CoV-2. The researchers constructed nanodecoys by fusing two types of cell membrane nanovesicles: (1) one derived from human embryonic kidney 293T cells that were genetically engineered to express SARS-CoV-2 receptor ACE2, and (2) another derived from human myeloid mononuclear THP-1 cells. The nanodecoys inhibited the replication and infection of SARS-CoV-2, and also suppressed immune disorder and lung injury in an acute pneumonia mouse model by neutralizing inflammatory cytokines, thus showing potential of nanotechnology against COVID-19 [150].

Li et al. [151] presented the anti-SARS-CoV-2 application of nanodecoys derived from human lung spheroid cells (LSCs). The study was done on SARS-CoV-2-infected cynomolgus macaque model, which when received nanodecoys via inhalation using a nebulizer showed a reduction of viral load and decrease in pulmonary fibrosis. Similarly, Zhang and coworkers [152] prepared two kinds of nanosponges (a type of lipid nanostructures) from cell membranes of either human lung epithelial type II cells or human macrophages. Because these nanosponges had on their surfaces protein receptors used by SARS-CoV-2 for cell entry, they could capture and neutralize SARS-CoV-2 and prevent the virus from infecting other host cells.

Recombinant S1 subunit protein from SARS-CoV-2 was encapsulated in cationic liposomes by Liu et al. [153] to prepare a nanovaccine. The vaccine elicited robust humoral responses in mice, and the serum from the vaccinated mice significantly inhibited SARS-CoV-2 infection in Vero cells. This lipid-based formulation technique presents an interesting approach that can be translated to clinical settings to tackle COVID-19.

Recently, nanobodies, which are highly stable small functional fragment of antibodies, are being used as therapeutic agents against many diseases. Canonical antibodies are comprise of two identical heavy and two identical light chains, both connected via interchain disulphide bonds and non-covalent interactions [154]. In comparison, nanobodies lack light chain and also the first constant domain (CH1) of the heavy chain, due to which nanobodies can attain compact structure and low dimensional size (4 × 2.5 × 3 nm) and low molecular weight (~15 kDa) than that of conventional antibodies [155],[156]. Nanobodies are naturally found in the camelid family (alpacas, camels, dromedaries, guanacos, and llamas) [157]. Nanobodies are highly stable and can easily be produced in large quantities in bacterial, yeast, or mammalian expression system [158]. Due to these reasons nanobodies are being utilized as therapeutic agents against SARS-CoV-2 infection (Table 5). Many studies in in vitro and in vivo conditions have showed strong neutralizing capability of nanobodies against SARS-CoV-2 even at nano molar and pico molar range of concentrations (Table 5). Nanobodies itself and their derived forms such as nanobody-coated nanostructures and nanobody-derived nanoprobes have successfully been utilized in imaging, target drug delivery and therapy, and diagnosis of diseases [159]. Therefore, nanobodies must be further explored for similar applications against SARS-CoV-2 to counter COVID-19 pandemic.

**Table 4.** mRNA-based vaccines using Lipid nanoparticles (LNPs) for the delivery of cargo.

Vaccine	Developer	Trial phase
ARCoV	Walvax Biotechnology Co., Ltd.; Abogen Biosciences Co. Ltd.; Yuxi Walvax Biotechnology Co., Ltd.	Phase 3
CVnCoV	CureVac; GSK	Phase 2b/3
DS-5670a	Daiichi Sankyo Co., Ltd.	Phase 1/2
MRT5500	Sanofi, Translate Bio	Phase 1/2
LUNAR-COV19 (ARCT-021)	Arcturus/Duke-NUS/Catalent	Phase 1/2
LNP-nCoVsaRNA	Imperial College	Phase 1/2
(Not named yet)	Shanghai Municipal Science and Technology Commission; Stemirna Therapeutics	Phase 1
(Not named yet)	Gritstone Oncology, Inc.; National Institute of Allergic and Infectious diseases (NIAID)	Phase 1
PTX-COVID19-B	Providence Therapeutics; Canadian government	Phase 1
ChulaCov19	Chulalongkorn University	Phase 1
D614G variant LNP Encapsulated RNA (Bancovid, Banagavax)	Globe Biotech Ltd	Pre-clinical
LNP-encapsulated mRNA encoding S	Max-Planck-Institute of Colloids and Interfaces	Pre-clinical
Comirnaty (BNT162b2)	Pfizer, BioNTech; Fosun Pharma	Authorized or approved
Moderna COVID-19 Vaccine (mRNA-1273)	Moderna, Biomedical Advanced Research and Development Authority (BARDA), National Institute of Allergic and Infectious diseases (NIAID)	Authorized or approved

**Source:** World Health Organization [116].

**Table 5.** Nanobodies against SARS-CoV-2.

Study	Findings	Effective inhibitory concentration
Xu et al. [160]	anti-RBD nanobodies isolated from llamas and from engineered mice neutralized SARS-CoV-2 variants	In picomolar range
Güttler et al. [161]	The nanobodies bound the open and closed states of the Spike protein and interacted tightly with RBD domain. The nanobodies were found to be highly thermo stable at 95 °C	17–50 pM
Sziemel et al. [162]	Recombinant alpaca antibodies neutralized live virus of B.1.351 variant of concern of SARS-CoV-2	IC_50_< 3nM
Custódio et al. [163]	Nanobodies, Sb23, isolated from a synthetic library, sybodies (Sb), targeted the receptor-binding domain (RBD) of the SARS-CoV-2 spike protein and able to neutralized pseudoviruse	IC_50_ of 0.6µg/mL
Ye et al. [164]	The nanobody Nanosota-1 can bound to the receptor-binding domain (RBD) of SARS-CoV-2 spike protein thus blocking out ACE2 binding to the RBD. Single dose of Nanosota-1 showed therapeutic efficacy in a hamster model of SARS-CoV-2 infection.	Neutralization dose 50% (ND_50_) of 0.16 µg/mL
Pymm et al. [158]	Nanobody cocktails administered prophylactically reduced viral loads and infection in mice challenged with the N501Y D614G SARS-CoV-2 virus variant	half-maximal inhibitory concentration (IC_50_) = 0.1 nM
Hanke et al. [165]	The alpaca derived nanobody, Ty1, prevented binding of SARS-CoV-2 RBD to its host cell receptor ACE2. The Ty1 nanobody can easily be expressed in bacteria with very high yield (>30 mg/L culture)	IC_50_ of 0.77 µg/mL (54nM)
Nambulli et al. [166]	Pittsburgh inhalable Nanobody 21 (PiN-21), which can be delivered intranasally, prevented and treated SARS-CoV-2 infection in Syrian hamsters	0.2 mg/kg
Esparza et al. [167]	NIH-CoVnb-112 nanobody blocked SARS-CoV-2 spike pseudotyped lentivirus infection of HEK293 cells expressing human ACE2	EC_50_ of 0.3µg/mL
Xiang et al. [168]	The nanobodies showed very high affinity (~10 pM) with RBD of spike protein and able to neutralize pseudotyped SARS-CoV-2	half-maximal inhibitory concentration as low as 0.058 ng/mL)
Koenig et al. [169]	Nanobodies able to neutralize SARS-CoV-2 and SARS-CoV-2–pseudotyped vesicular stomatitis virus	IC_50_ value of 60 nM
Lu et al. [170]	Nanobodies Nb91-hFc and Nb3-hFc against spike protein and its RBD domain neutralized spike pseudotyped viruses in vitro	IC_50_ of 1.54 nM

## 5. Conclusions

The COVID-19 pandemic has created unprecedented challenges for humanity. Nanomaterials present interesting candidates that can be incorporated into therapy and diagnostics for SARS-CoV-2. Nanomaterials, such as metal nanostructures, graphene and graphene derivatives, nanocomposites, and lipid-derived nanomaterials have spawned novel detection methods for COVID-19. Similarly, to prevent spread of SARS-CoV-2 in public places and healthcare settings various new products (face masks and other personal protective clothings, surface coatings, etc.) based on nanotechnology have emerged. However, the claim of such products in controlling the spread of SARS-CoV-2 must be properly verified by the appropriate agencies of the respective countries to strengthen the fight against COVID-19. Similarly, if any such product is using metal nanostructures, then the toxicity analysis of metal nanostructures on human health must be properly assessed. All over the world, many research groups are also utilizing nanotechnology for designing vaccines and therapeutic agents against SARS-CoV-2. But these efforts have to keep pace with deadlier SARS-CoV-2 variants that are continuously emerging from different parts of the world. Further, the findings of research studies that used nanotechnology-based approaches against SARS-CoV-2 in lab settings need to be translated to clinical settings in humans. This present an attractive opportunity for the scientist working in the field of nanotechnology to use their expertise for fighting the COVID-19 pandemic by tooth and nail.
